# Supplementary material for: Recruiting, training and managing a sensory panel in odor nuisance testing
Source: PLoS One. 2021 Oct 19;16(10):e0258057. doi: 10.1371/journal.pone.0258057 (PMC8525733; doi:10.1371/journal.pone.0258057)
Supplement: Attachment — Submitted filename: PLOS ONE REVIEW REPORT .docx [file pone.0258057.s002.docx]

**PLOS ONE**

**REVIEW REPORT**

**Manuscript Number:	PONE-D-21-12936**

**Title:	Recruiting, Training and Managing a Sensory Panel in Odor Nuisance Testing**

The paper shows a good suggestive work which is essential for Odor Nuisance Testing purpose. It is recommended for publication subjected to following minor revisions.

1. In the first paragraph of the introduction section, the authors are advised to mention more details about PN-EN 13725:2007 standard for more clarity.

2. Further,the authors advised to mention correct section heading numbers, like 3 for Final selection (Qualification) in place of 2.

3. The quality of figures arenot good. Hence authors are advised to show better resolutions in the paper.

4. The authors should also cite few more latest referred papers.

**Recommendation**… To be accepted after minor revision.

**Shankar Swarup Das**

**Reviewer**

**Dated.27.06.2021**
