# Supplementary material for: Recruiting, training and managing a sensory panel in odor nuisance testing
Source: PLoS One. 2021 Oct 19;16(10):e0258057. doi: 10.1371/journal.pone.0258057 (PMC8525733; doi:10.1371/journal.pone.0258057)
Supplement: Attachment — Submitted filename: Manuscript Number PONE-D-21-12936 _PlosOne-Review comments.doc [file pone.0258057.s003.doc]

Manuscript Number: PONE-D-21-12936 Full Title: **“Recruiting, Training and Managing a Sensory Panel in Odor Nuisance Testing**”; Research Article

**Reviewer comments**

Overall this research objective is good and author tried to do some Odor Nuisance Testing, But there are some points to be considered before publishing this papers

1-Authors focused to Recruitment and Final selection (Qualification) approaches on panelist members based on duration of training periods that can do odor nuisance testing. But, there is no proper proof on scientific basis that can ensure for different panels for same food or non-product odor. So, author has to put some efforts/ evidences on this query

2-What are software used for creating these figure and how this odor testing procedure can be suitable for other products that contained many odor compound. This paper only mentioned few examples like n-butanol.

3-There are many old references, that is not suitable for current form of this paper, Go for new references that can support this objects

After adding these suggestions, paper can go for publication without sending to reviewer.
